# The Role of Empathy in ADHD Children: Neuropsychological Assessment and Possible Rehabilitation Suggestions—A Narrative Review

**DOI:** 10.3390/medicina61030505

**Published:** 2025-03-15

**Authors:** Antony Casula, Giulia Belluardo, Carmine Antenucci, Federica Bianca, Francesco Corallo, Francesca Ferraioli, Domenica Gargano, Salvatore Giuffrè, Alice Lia Carmen Giunta, Antonella La Torre, Simona Massimino, Alessio Mirabile, Giuliana Parisi, Cono Daniele Pizzuto, Maria Cristina Spartà, Alessia Tartaglia, Francesco Tomaiuolo, Laura Culicetto

**Affiliations:** 1 Department of Cognitive Sciences, Psychology, Education and Cultural Studies, University of Messina, 98122 Messina, Italy; antonycasula@gmail.com (A.C.); frferraioli@unime.it (F.F.); simona.massimino@unime.it (S.M.); 2 International School of Advanced Studies, Center for Neuroscience, University of Camerino, 62032 Camerino, Italy; alice.giunta@unicam.it; 3 Department of Human Sciences, University Guglielmo Marconi of Rome, 00193 Rome, Italy; g.belluardo@unimarconi.it; 4 MIM—School of Employment “Cosimo Ridolfi” Comprehensive Institute, 66050 Monteodorisio, Italy; carmine.antenucci@studenti.unime.it; 5 Masterclass in “Neuropsychological Intervention Disorders and Technologies. From Childhood to Adolescence”, University of Messina, 98122 Messina, Italy; federicabiancafb90@gmail.com (F.B.); sergiuffre@gmail.com (S.G.); antlatorre@unime.it (A.L.T.); alessiatartaglia1998@gmail.com (A.T.); 6 IRCCS Centro Neurolesi Bonino Pulejo, 98125 Messina, Italy; alessio.mirabile@irccsme.it (A.M.); laura.culicetto@irccsme.it (L.C.); 7 IISS Alessandro Volta, Centro Ateneo per la Formazione Degli Insegnanti–Università Degli Studi di Palermo, 90128 Palermo, Italy; garganodomenica27@gmail.com; 8 Dipartimento di Medicina Clinica e Sperimentale, Università di Messina, Piazza Pugliatti, 98122 Messina, Italy; giulianaparisi99@yahoo.com (G.P.); francesco.tomaiuolo@unime.it (F.T.); 9 I.C. Maneri Ingrassia Don Milani, Università Degli Studi di Palermo, 90128 Palermo, Italy; pizzutocono@gmail.com; 10 “L. & V. Pasini” Technical and Technological Institute, 36015 Schio, Italy; mariacristina.sparta@gmail.com

**Keywords:** ADHD, theory of mind, empathy, neuropsychological assessment

## Abstract

*Background and Objectives:* Theory of mind (ToM) deficits in children with ADHD are closely related to social difficulties and problems in interpersonal interactions. Evidence suggests that these cognitive deficits negatively affect the ability to understand and respond to others’ emotions and intentions, thus contributing to social isolation and a lower quality of life. However, the findings across studies vary, indicating that ADHD subtype and comorbidities, such as anxiety and mood disorders, can significantly influence sociocognitive deficits, modulating the extent of social problems. *Materials and Methods:* This review examines the relationship among ADHD, ToM, and empathy, analyzing studies comparing children with ADHD with peers with typical development or other neurodevelopmental conditions. A search in PubMed, Scopus, and the Cochrane Library prior to January 10, without time restrictions, using “ADHD”, “Cognitive Empathy”, and “Theory of Mind” identified relevant studies assessing these abilities through neuropsychological tests or questionnaires. *Results:* Of the initial 243 studies, 23 studies met the inclusion criteria. Children with ADHD exhibited significant impairments in ToM and empathy, affecting social cognition and interpersonal understanding. Various assessment tools revealed difficulties in understanding beliefs, emotions, and intentions, with executive function deficits playing a crucial role in shaping these social challenges. *Conclusions:* This review highlights the need for targeted therapeutic interventions that not only address cognitive deficits but consider emotional and metacognitive aspects, such as emotion regulation and self-awareness. Future research should focus on integrating executive function training with approaches that develop metacognitive and emotional skills, thus providing more comprehensive support.

## 1. Introduction

Attention deficit hyperactivity disorder (ADHD) is a neurodevelopmental condition with onset typically in childhood. It is characterized by a spectrum of symptoms that include difficulties in sustained attention, impulsive behavior, and excessive activity levels. Although ADHD was first described as early as 1775, our understanding of the disorder remains in a continuous state of development [1,2]. Cognitive and social functioning deficits are often associated with motor abnormalities, such as neurological subtle signs (NSS), which are markers of atypical neurodevelopment and predictors of the severity of functional impairment in children with ADHD [3]. Moreover, the emotional sphere plays a central role in the clinical profile of ADHD. Many individuals with this disorder struggle with emotion regulation, exhibiting intense emotional reactions, low stress tolerance, and heightened sensitivity to frustration. These difficulties can exacerbate cognitive and social deficits, negatively impacting interpersonal relationships and contributing to the development of anxiety and depression [1,3]. Emotional challenges appear to be linked to specific neurobiological abnormalities, particularly in regions such as the limbic system and the prefrontal cortex, which are crucial for modulating emotional responses. Alterations in these areas may impair the ability to integrate and regulate emotions, leading to excessive reactions and increased vulnerability to stress [4]. The developmental trajectory of ADHD, with its associated risks such as low academic achievement, poor school performance, behavioral problems, and difficulties in social and family relationships, has significant societal implications. These challenges can lead to long-term issues, including higher rates of delinquency, substance abuse, and difficulties in maintaining stable employment and relationships in adulthood [4]. As a result, ADHD not only affects individual quality of life but imposes broader social and economic costs, including increased demands on the educational, healthcare, and legal systems. Addressing ADHD through early diagnosis, intervention, and support is crucial for mitigating these societal burdens and promoting better long-term outcomes for affected individuals.

ADHD is a complex, multidimensional disorder, the etiology of which is attributed to the interplay of genetic, biological, and environmental factors [5].

For instance, a recent meta-analysis conducted in a sample of 38,691 individuals with ADHD and 186,843 TD reported 76 potential risk genes for ADHD linked to early brain development, especially in frontal cortex [6].

Several environmental risk factors have been linked to the development of ADHD, including prenatal maternal stress [7], maternal smoking during pregnancy [8], prenatal maternal inflammation [9], preterm birth and/or low birth weight [10], exposure to social disadvantage and adversity [11], average levels of lead exposure [12], and maternal prepregnancy body mass index (BMI) [13]. These factors interact with genetic predispositions and contribute to the risk of developing ADHD, further highlighting the multifactorial nature of the disorder [14].

The ADHD criteria as identified by the Diagnostic and Statistical Manual of Mental Disorders fifth edition (DSM-5-TR) (American Psychiatric Association, 2022 [15]) are described in Table 1: 

ADHD is a heterogeneous disorder with varied clinical shapes, previously termed “subtypes” in DSM-IV and now referred to as “presentations” in the DSM-5. The Diagnostic and Statistical Manual of Mental Disorders, Fifth Edition (DSM-5), identifies three presentations of ADHD (American Psychiatric Association, 2013 [16]):

The first presentation is the predominantly inattentive presentation (ADHD-IA/PI), characterized by difficulties with sustaining attention, organization, following instructions, and completing tasks. Individuals with ADHD-PI may appear forgetful, easily distracted, and have difficulty following through on activities. 

The second one is the predominantly hyperactive/impulsive presentation (ADHD-HI), in which children exhibit six or more symptoms of H/I but fewer than six symptoms of IA. They are marked by excessive motor activity, restlessness, difficulty remaining seated, interrupting others, and difficulty waiting their turn. Individuals with ADHD-HI may fidget, squirm, talk excessively, and have difficulty controlling their impulses. 

The third one is the combined presentation (ADHD-C), where children meet the criteria for six or more symptoms in both inattention and hyperactivity/impulsivity dimensions (American Psychiatric Association, 1994 [17]). ADHD-C is the most common presentation of ADHD [18].

Despite clinical efforts to differentiate between ADHD presentations, the validity of this subdivision has been challenged by experimental findings. For instance, Chabildas and collaborators suggest that all three presentations share a core attention deficit, which may account for most of the other cognitive and behavioral impairments observed, calling into question the distinctiveness of the presentations [19].

Meta-analyses indicate that the global prevalence of ADHD in children and adolescents is approximately 7.2%, with variations across regions and methodologies [20]. ADHD is consistently found to be more prevalent in males than females, with a male-to-female ratio ranging from 2:1 to 4:1 [18]. While historically considered a childhood disorder, ADHD persists into adulthood for a significant proportion of individuals, with an estimated prevalence of 2.5% in adults [21].

Higher prevalence rates are reported in North America and Africa, while lower rates are observed in Asia and South America [22]. These variations may reflect differences in cultural attitudes towards ADHD, diagnostic practices, and access to mental health services.

Diagnosing ADHD can be challenging because of the subjective nature of the symptoms and the frequent overlap with other conditions. Accurate diagnosis requires a comprehensive evaluation by a qualified mental health professional, including a detailed clinical interview, behavioral observations, and standardized rating scales [17].

The treatment of ADHD typically involves a multimodal approach, including:

Pharmacological interventions: Stimulant medications (methylphenidate, amphetamines) are often the first-line treatment for ADHD, but nonstimulant medications (atomoxetine, guanfacine) may also be effective [23].

Behavioral therapies: Parent training in behavior management, classroom interventions, and social skills training can help individuals with ADHD manage their symptoms and improve their functioning [24].

Psychoeducation: Providing information and support to individuals with ADHD and their families is crucial for understanding the disorder and developing effective coping strategies [25].

ADHD is a complex and heterogeneous disorder with significant implications for individuals, families, and society. Understanding the prevalence, clinical presentation, and challenges associated with ADHD is essential for promoting early identification, effective intervention, and improved quality of life for those affected by this condition. Numerous theories have been developed to explain the origins and nature of deficits associated with ADHD. A few key examples, though not an exhaustive list, include:

Dual Pathway Models: Nigg [26] proposed that ADHD arises from both cognitive and emotional pathways, suggesting that impairments in executive functions (EFs) and emotional regulation contribute to the behavioral symptoms associated with ADHD [26].

Executive Functioning Model: Barkley [27,28] hypothesized that ADHD arises from a core deficit in executive functions, which are crucial for self-regulating social interactions. According to Barkley, impaired inhibitory control triggers deficits in other EFs, such as working memory, emotional regulation, and cognitive flexibility, resulting in challenges in self-regulation and the ability to produce self-directed behaviors. This cascade effect highlights inhibitory control as central to the broader EF impairments observed in individuals with ADHD [29].

Milic et al. [30] hypothesized that the combined subtype and the predominantly inattentive subtype are distinct and unrelated disorders. The hypothesis comes from the observation of differences between the two groups in demographics, measures of cognitive and neuropsychological functioning, family history, treatment response, and prognosis. However, the hypothesis has faced criticism, as the assumption of no similarities between the inattentive and combined subtypes has been repeatedly questioned in various studies [31,32].

Some experimental results have pointed out that ADHD children, if compared with typical-development children, show deficits in some tasks related to ToM (theory of mind), such as false belief and “faux pas”. ToM is the ability to recognize and attribute to others mental states, such as beliefs, desires, and intentions, which may differ from one’s own. This capacity is essential for understanding others’ behaviors and for effective interaction [33,34]. Additionally, difficulties in understanding pragmatic language and a high error rate in attribution of mental states and emotions based on looks were observed [29,33].

These difficulties can be traced to specific mechanisms of empathy in ADHD. While affective empathy, that is, the ability to feel emotions in response to those of others, appears to be relatively preserved, cognitive empathy, that is, the ability to understand and interpret the mental states of others, is impaired. As a result, children with ADHD struggle to pick up complex social cues, which may adversely affect their interpersonal interactions and relationships [25].

A systematic review by Pineda-Alhucema et al. [29] sought to integrate the hypotheses of EF deficits [34] and ToM deficits in children with ADHD. They hypothesized that deficits in EFs may be linked to impaired ToM development in these children. Their review found a positive correlation between EF and ToM deficits in the majority of studies examined (six out of eight); however, the limited number of studies with available statistical data prevented firm conclusions.

The link between EFs and ToM in individuals with ADHD has been highlighted by recent research: deficits in working memory and ToM may contribute to the social perception challenges seen in children with ADHD, with findings showing that these children experience greater difficulty in interpreting biological motion in noisy environments and perform more poorly on ToM tasks than both children with specific learning disorders and TD peers [35,36].

ToM is distinct from empathy: empathy refers to the ability to recognize, understand, and share the emotions of others. It involves not only a cognitive understanding of others’ emotional experiences but an emotional response to those experiences [37].

The neural mechanisms involved in empathy include the prefrontal cortex, associated with emotion regulation and social judgment; the anterior cingulate cortex, involved in emotion management and the processing of others’ emotional experiences; the insula, engaged in the perception of emotions and subjective experiences of pain and discomfort; and pain neural systems, activated even when observing others in pain, contributing to our empathic response [38].

Several disorders are associated with deficits in ToM: autism spectrum disorder (ASD), Asperger syndrome, schizophrenia, personality disorders, and brain injuries [39,40,41].

This narrative review aims to provide a comprehensive overview of proposed ToM deficits in children with ADHD by analyzing experimental studies that have investigated this hypothesis through comparisons with TD controls. The review will address limitations in the existing studies and offer recommendations for future research directions.

## 2. Materials and Methods

### 2.1. Search Strategy

Relevant studies were identified through searches of the PubMed, Scopus, and Cochrane databases published before 10 January with no time limit. The search combined the following terms using appropriate Boolean operators (i.e., AND, OR): “ADHD”, “Cognitive empathy”, “Theory of mind.” The same queries were applied across all databases.

### 2.2. Inclusion and Exclusion Criteria

Studies meeting the following criteria were included in the review:

1. The study population included ADHD patients;
2. Theory of mind or cognitive empathy was assessed using questionnaires;
3. Articles were published in English.

Studies were excluded if they met any of the following criteria:

4. Reviews or meta-analyses;
5. Conference papers or editorials;
6. Duplicated studies;
7. Animal studies.

There were no restrictions on the year of publication for the studies considered.

### 2.3. Study Selection

After removing duplicates, all articles were evaluated and selected based on title, abstract, and full text. The evaluation process, conducted using Microsoft Excel (Microsoft Corp., Redmond, WA, USA), was completed in three rounds, with each study being “double-checked” for inclusion by 13 researchers.

**Figure 1:**
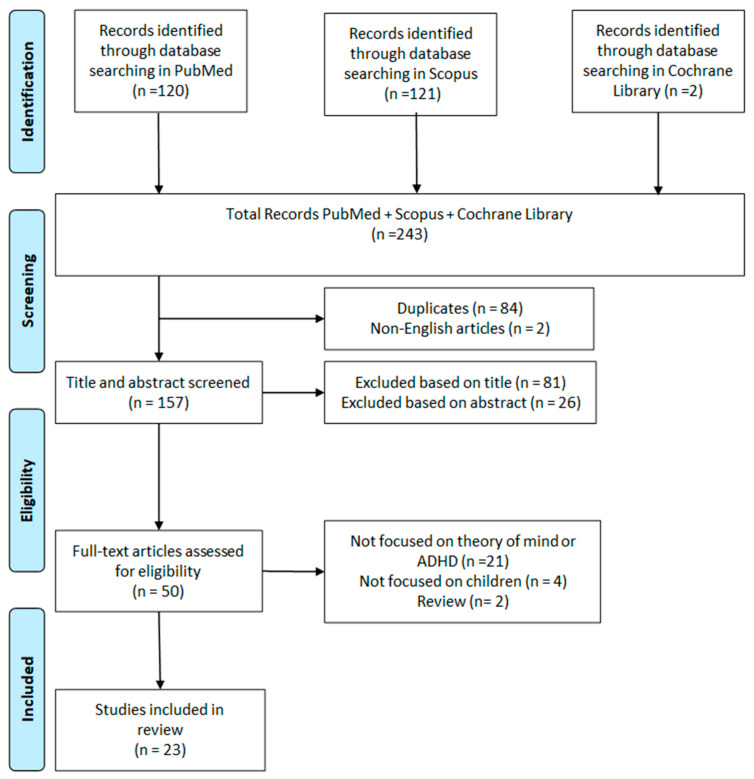
Search and selection of eligible articles.

We identified a total of 243 studies: 120 articles from PubMed, 121 from Scopus, and 2 from the Cochrane Library. After eliminating 84 duplicate studies and 2 non-English studies, 159 publications were initially selected. Following a full-text review and the application of predefined inclusion criteria, 81 articles were excluded based on the title, 26 based on the abstract, 21 because they did not focus on empathy or theory of mind, 4 because they were not related to children, and 2 because they were reviews. After a thorough evaluation of the remaining manuscripts, 23 articles satisfied the inclusion and exclusion criteria (Figure 1).

## 3. Results

Empathy is a multifaceted construct often assessed using a variety of tests that examine different aspects of social cognition, including ToM. In this review, the findings have been organized based on the specific tests administered to measure empathy and related constructs in children with ADHD. This approach allows for a clearer understanding of the specific strengths and weaknesses revealed by each assessment tool, highlighting the nuanced ways in which ADHD impacts social cognition and interpersonal understanding. Below, we present the results for each test, providing insights into the unique contributions and limitations of these measures in capturing ToM abilities and empathy deficits in children with ADHD (Table 2).

We also present a table with the strengths and limitations of the tests used in this review in Table 3.

### 3.1. False Belief Task (FBT)

Among the studies reviewed, false belief tasks (FBT; [62]) emerged as a widely used tool for measuring ToM abilities in children with ADHD. These tasks revealed consistent impairments in both first- and second-order ToM, concerning a person’s ability to understand that others have mental states different from their own and the ability to understand that a person may have beliefs or thoughts about what another person believes or knows, respectively. Bolat et al. [58] reported significantly lower ToM performance in both first- and second-order FBT in a sample of 69 ADHD children compared with 69 TD control children aged 8–15 years. Sevincok et al. [43] extended these findings in a sample of 50 ADHD and 40 TD children aged 8–14 years, with the results consistently indicating poorer performance in the ADHD group. Caillies et al. [48] evidenced reduced performance on a second-order false belief task in 15 ADHD children aged 7–10 years, further reinforcing these findings. Conversely, Löytömäki et al. [42] reported significantly lower ToM performance in a study of 17 ADHD and 106 TD children aged 6–10 years in the Sally and Anne task (first-order) but not in the Ice Cream Van story (second-order). As Löytömäki et al. [42], Kilincel et al. [46] found reduced second-order FB performance in ADHD children (N = 42) compared with TD children (N = 41), yet not reduced first-order performance.

Furthermore, Perner et al. [60] examined children at high risk for ADHD, finding similar no significant deficits in advanced ToM (i.e., second-order false belief tasks) despite observable impairments in executive functioning (EF) tasks such as planning, attention, and inhibitory control. This suggests that ToM development in children with ADHD may not be as closely linked to EF as previously assumed. Similarly, Yang et al. [59] did not find any ToM difference between ADHD (N = 26) and TD (N = 30) children ranging from 3.3 to 13.5 years old in FBT tasks (composite score). Conversely to what was hypothesized by the authors, ADHD children did not show any EF impairments compared with TD controls.

Overall, the findings have revealed consistent ToM impairments in children with ADHD as measured by first-order false belief tasks, though some studies have indicated inconsistent results on second-order ones, as well as nuanced variations in the relationship between ToM and EFs in this population.

### 3.2. Faux Pas Recognition Test (FPRT)

The Faux Pas Recognition Test (FPRT) has consistently demonstrated its utility in evaluating social cognition and ToM deficits among individuals with ADHD, providing insights into their social and cognitive challenges [63]. Dağdelen [47] examined ToM impairments in 60 adolescents (12–16 years old) with ADHD, 60 with autism spectrum disorder (ASD), and 60 TD controls, finding that both ADHD and ASD groups scored significantly lower than TD participants in the FPRT. However, the ToM impairments were notably more severe in the ASD group compared with the ADHD group, underscoring a gradient of social cognitive deficits. This suggests that while ADHD impairs ToM, the deficits may not be as profound as in ASD. Similarly, Kilincel et al. [46] and Mary et al. [33] extended these findings, revealing significant difficulties in recognizing faux pas in samples of 42 and 22 ADHD children and 41 and 22 TD controls, respectively. It is worth noting that Kilincel et al. [46] tested adolescents in an age range of 12–16, while Mary et al. [33] tested preadolescent (8–12) children, evidencing that the impairments follow the children in different evolutionary phases. Unlike previous studies, Kilincel et al. [46] explicitly linked the severity of ADHD symptoms to the extent of ToM deficits, emphasizing the role of ADHD-related inattention and impulsivity in social cognitive challenges. In contrast, Maoz et al. [44] explored both empathy and ToM in children with ADHD, offering a complementary perspective. Using the FPRT and the Interpersonal Reactivity Index (IRI), the study highlighted significant ToM impairments and cognitive empathy deficits, particularly in perspective-taking and fantasy. Interestingly, Maoz et al. [44] observed that ADHD symptoms correlated negatively with cognitive empathy scores. Moreover, the administration of methylphenidate (MPH) led to marked improvements in FPRT performance, aligning the ADHD group’s results with those of healthy controls [44]. This suggests that executive function deficits, such as attention and inhibition challenges, play a pivotal role in ToM impairments, and that MPH may mitigate these difficulties. Conversely, Bozkurt et al. [51] did not find any statistically significant impairment in FPRT scores in 47 ADHD children (range age: 8–13) as compared with 38 TD controls.

### 3.3. Happé’s Strange Stories

The Happé’s Strange Stories task, a measure of advanced ToM [64], was employed by Parke et al. [50]. The study consistently demonstrated that children with ADHD exhibited significant difficulties in understanding complex social interactions compared with their TD peers. Parke et al. [50] assessed a sample of 25 children with ADHD and 25 TD children aged 7–13 years, finding that ADHD children scored significantly lower on the task, indicating challenges in interpreting subtle social scenarios and intentions.

### 3.4. Narrative and Internal State Language (ISL): Telling a Story from a Book

The ability to construct coherent narratives and use mental-state language (named as internal state language, or ISL [65]) was assessed by Rumpf et al. [53] in a study comparing children with Asperger syndrome (AS), ADHD, and a TD group, all aged 8–12 years. Despite similar syntax and cohesion across groups, children with AS and ADHD produced shorter, less coherent narratives than the TD group. Furthermore, children with AS, regardless of ADHD comorbidity, used fewer pronouns and references to mental states, reflecting limitations in their use of internal state language (ISL). These findings suggest that while grammatical skills may be intact, narrative coherence and the ability to express internal states remain areas of difficulty for children with AS and ADHD.

### 3.5. Developmental Neuropsychological Assessment, Second Edition (NEPSY-II)

The NEPSY-II [66] was employed in three studies to assess ToM and related affective abilities in children with ADHD. The studies collectively explored the relationship between ADHD and ToM, emphasizing the role of EF in shaping social cognition and empathy. Berenguer et al. [61] highlighted moderate ToM deficits in children with ADHD compared with TD peers, although these children performed better than those with ASD. The deficits were especially prominent in real-world social contexts, where ADHD children struggle to comprehend others’ emotions, intentions, and mental states. Furthermore, inattention—a hallmark symptom of ADHD—was strongly associated with impaired EF processes and ToM abilities, indicating that disrupted attention regulation negatively impacted social interaction and empathy.

Parke et al. [50] found that while children with ADHD did not differ significantly from TD peers in the NEPSY-II Contextual task for affective ToM, they exhibited marked difficulties in the Affect Recognition subtest. This reflects challenges in recognizing emotional expressions, a critical component of social cognition. Similarly, Pitzianti et al. [3] used the NEPSY-II to assess ToM and emotion recognition in 23 ADHD children and 20 TD controls. Their findings suggested variability in ToM and emotion recognition performance, with ADHD children not consistently showing deficits when compared with TD peers. This variability underscores that socioemotional difficulties in ADHD may depend on individual differences and contextual factors, rather than being uniform across the population.

### 3.6. Reading Mind in the Eyes Test (RMET)

The Reading Mind in the Eyes Test (RMET; [67]) has been widely used to assess ToM abilities, particularly in emotion recognition, among children and adolescents with ADHD. Several studies reported significant differences between ADHD and TD groups. For example, Sevincok et al. [43] found that 50 children with ADHD (8–14 years old) obtained lower RMET scores than 40 TD children. Özbaran et al. [45] and Yilmaz Kafali et al. [55] replicated these findings with 100 ADHD and 100 TD children and with 60 ADHD and 60 TD children, respectively, in an age range of 11–17 years old. Dağdelen (2021) confirmed and extended the same results in a sample ranging from 12 to 16 years old. Bozkurt et al. [51] demonstrated that children with ADHD (8–13 years old) exhibited lower accuracy in recognizing facial expressions of disgust and anger compared with TD children. Moreover, ADHD participants in the study showed reduced fixation on the eye region during an eye-tracking task, suggesting altered visual attention to facial cues, although no significant correlation was found between these fixations and ADHD symptoms or ToM test scores. Conversely, a study by Razjouan et al. [49] did not find meaningful disparities in RMET scores among 52 ADHD and 41 TD adolescents aged 12–18 years. Other investigations provided insights into broader neurodevelopmental contexts. For instance, Demurie et al. [56] identified impairments in perspective-taking abilities among adolescents with ADHD and ASD, noting that ADHD participants scored lower than controls on both static and naturalistic ToM tasks. These findings align with research by Yilmaz Kafali et al. [55], previously introduced), who explored ToM impairments in adolescents with ADHD involved in bullying behaviors. Their study linked poorer RMET performance to a higher likelihood of both victimization and perpetration. Emotional dysregulation, assessed through the Difficulties in Emotion Regulation Scale (DERS), was particularly evident among perpetrators, highlighting the interplay between ToM deficits, emotional regulation challenges, and social difficulties. Collectively, these studies underscore the complexity and heterogeneity of ToM impairments in ADHD, influenced by factors such as task type, emotion-specific challenges, and broader cognitive and emotional processes. These impairments contribute to difficulties in managing impulsivity and goal-directed behavior during emotional distress. The findings underscore the interplay between ToM and ER challenges, suggesting that ToM deficits may partly underlie emotional dysregulation in ADHD, ultimately impacting social interactions and cognitive empathy.

### 3.7. Theory of Mind Assessment Scale (Th.o.m.a.s.)

The Theory of Mind Assessment Scale (Th.o.m.a.s.; [68]), has been used to evaluate ToM abilities and EF in children with ADHD. Razjouan et al. [49] found no significant differences between children with ADHD and their TD peers (aged 12–18 years), indicating comparable ToM performance. 

### 3.8. Animated Triangles Task—Paradigmatic Task of Moving Forms

The Animated Triangles Task [69] provided additional evidence of ToM and EF impairments. Mohammadzadeh et al. [57] observed significant differences in performance between ADHD and control groups in a sample of 30 ADHD children and 30 TD children aged 7–9 years. ADHD participants demonstrated poorer scores on the Animated Triangles Task, highlighting challenges in attributing mental states to abstract moving shapes. Notably, while ToM deficits were evident, they were not directly correlated with EF impairments. However, deficits in cognitive flexibility and inhibitory control, as measured by Intra–Extra Dimensional Set Shift (IED) tasks, were shown to influence ToM performance.

Building on these insights, Mohammadzadeh et al. [54] utilized the moving shapes paradigm to investigate intentional behavior understanding in ADHD children. Findings revealed that ADHD participants used fewer mental state terms, offered less appropriate descriptions of interactions, and were more likely to use negative emotional terms (e.g., anger, fear) compared with TD children, who predominantly used positive terms such as happiness and joy. This indicates not only cognitive deficits in mental state attribution but emotional biases in social interpretation.

These studies collectively highlight the complex interplay of ToM and EF impairments in ADHD, emphasizing the unique challenges these children face in social–cognitive processing across diverse contexts.

### 3.9. Empathy Quotient (EQ)

The Empathy Quotient (EQ) [41] is a self-report questionnaire developed to address the lacks in previous empathy scales. Lee et al. [52] investigated structural differences and cognitive and affective empathy in adolescents with ADHD and TD controls. Their findings indicated that individuals with ADHD exhibited impairments in cognitive empathy, particularly in perspective-taking, while emotional empathy remained relatively intact. Structural brain differences in ADHD included reduced cortical volume in regions associated with emotional empathy (e.g., posterior insular and supramarginal cortices) and increased nucleus accumbens volume, potentially influenced by stimulant medication. Unlike in TD peers, no correlation was found between empathy measures and brain structure in individuals with ADHD. These findings underscore the role of neuroanatomical alterations in ADHD-related social and empathy deficits [52]. 

### 3.10. Theory of Mind Inventory, ToM Task Battery

Singh et al. [35] examined ToM and EF in children with ADHD, specific learning disorder (SLD), and TD peers by employing the ToM Inventory. The study found that children with ADHD showed significant deficits in ToM, including early, basic, and advanced ToM skills, compared with the TD and SLD groups. These deficits, observed in tasks such as understanding emotions and second-order beliefs, contribute to challenges in empathy and social interactions. To assess these abilities, the researchers used the ToM Inventory (completed by caregivers) and the ToM Task Battery, which evaluated false beliefs and emotional understanding.

### 3.11. ToMI

The Theory of Mind Inventory (ToMI; [70]) is a neuropsychological assessment instrument designed to measure theory-of-mind (ToM) skills in individuals, focusing specifically on how they understand and interpret the thoughts, feelings, and intentions of others. It was developed as a revised version of the Perceptions of Children’s Theory of Mind Measure–Experimental version (CToMM-E) to measure parent-reported ToM. By employing this task, the study performed by Berenguer et al. [61] investigated the relationships among ADHD, ASD, EF, and ToM in the context of social interactions in a sample of 30 ASD, 35 ADHD, 22 ASD + ADHD, and 37 TD children aged 7–11 years old. Children with ADHD displayed significant challenges in social cognition, characterized by lower ToM abilities compared with TD peers. Additionally, ASD and ASD + ADHD showed poorer ToM performance compared with both TD and ADHD groups. These deficits were evident across either early, basic, and advanced ToM skills, including difficulties with understanding emotions, making complex social judgments, and interpreting second-order inferences. Importantly, while ToM impairments were identified, the study highlighted that these challenges might stem from deficits in EF, such as behavioral regulation and inhibition. The findings suggest that EF impairments, particularly in response inhibition, play a central role in the social difficulties experienced by children with ADHD. These EF deficits hinder the application of ToM skills in everyday social situations, contributing to peer relationship problems and lower social acceptance. However, ToM did not emerge as a direct mediator in the relationship between ADHD symptoms and social challenges, emphasizing the complexity of the interplay among EF, ToM, and social outcomes. Overall, the study underscores the critical role of EF impairments in shaping the socioemotional difficulties of children with ADHD, with ToM deficits further compounding these challenges in social contexts [61].

## 4. Discussion

While the results of this review are very promising, they also highlight the difficulties still present in outlining a clear and universal framework for the assessment and treatment of ToM issues in children with ADHD. Although studies have provided important insights into how ToM deficits affect the social and cognitive abilities of these children, the lack of a shared model for the assessment and design of rehabilitation interventions remains a significant challenge. Research in this field is still in the early stages of development, and the variables to be considered are multiple and often not fully explored [71].

One of the most critical aspects emerging from the evidence is the difficulty in identifying a common guideline or systematic approach that can be uniformly applied to all children with ADHD. Although recurring patterns of ToM deficits associated with ADHD have emerged, research to date has failed to define a clear and valid set of criteria for designing targeted interventions. Each child with ADHD presents a unique combination of difficulties, which varies not only according to symptom severity but in relation to other individual factors such as family background, school environment, and psychological comorbidities. This variability makes it difficult to apply a universal treatment model [72].

Furthermore, current research methodologies have led to a breakdown of problems by patient groups and specific domains, but these domains are not always sufficiently defined or comprehensive. For example, first-order ToM deficits have been identified, which relate to understanding the basic beliefs and intentions of others, as have second-order deficits, which involve a more complex and multilevel understanding of social interactions. However, such distinctions may not capture the full complexity of the difficulties that children with ADHD encounter in their social and cognitive development. Emotional and metacognitive dimensions, for example, are still poorly explored, and may prove to be crucial to a deeper understanding of these children’s social interaction difficulties [73].

Another crucial aspect concerns the integration of different domains of functioning. Problems related to ToM are not isolated but closely linked to difficulties in other cognitive and emotional domains, such as emotion control, attention regulation, and the management of impulsive behaviors. Research has shown that deficits in EFs, such as impulsive response inhibition, working memory, and cognitive flexibility, underlie many of the ToM deficits in children with ADHD. However, the link between EFs and ToM is complex and still not fully understood. An approach that does not consider this interconnection risks not fully addressing the difficulties these children face [74].

Therefore, the need for a personalized approach becomes increasingly evident. Each child with ADHD has a unique profile that expresses itself in different ways and with varying intensity. While some children may have greater difficulties in understanding the emotions of others, others may be more oriented towards problems of social impulsivity, such as responding inappropriately to social cues. Therefore, it is crucial to adopt a treatment model that is not limited to a standardized approach but takes into account the specific needs of each child. This may require a highly individualized assessment process that considers, in addition to ToM difficulties, other factors, such as emotional regulation, metacognitive abilities, and the presence of any other comorbidities, such as anxiety or depression, that might affect a child’s ability to relate to others [75].

The customized approach should also be flexible and able to adapt to children’s changing circumstances during the course of treatment. Children with ADHD develop heterogeneously, and their difficulties may evolve over time. Some may develop more advanced social skills as they improve in executive function control, while others may need more targeted and intensive interventions to address specific social or emotional deficits. In this context, ongoing assessment and monitoring of progress are essential to adapt treatment to the needs that emerge during the course of treatment [76].

In summary, although research on ToM in children with ADHD has made significant progress, there are still many unresolved issues that require further attention. Establishing a systematic approach for the assessment and design of rehabilitation interventions remains a challenge, especially because of the great variability in the profiles of children with ADHD. To address these difficulties, it is essential to develop therapeutic models that are as flexible as they are personalized in order to respond adequately to the complexity of the difficulties that characterize this condition. The future of research in this field will have to focus on the integration of cognitive, emotional, and social aspects, trying to develop interventions that improve not only ToM skills but other crucial areas such as emotional regulation and control of EFs, for global and more effective treatment of the disorder [77].

One of the most commonly used instruments to assess ToM in children is the FBT. This test investigates a child’s ability to understand that others may have beliefs that differ from their own and from reality. Although the FBT provides useful information, its application in the context of ADHD has shown that children with this condition score lower than their peers with typical development (TD), with a particular difficulty in more complex situations such as second-order situations. These findings have been confirmed by several recent studies, including those of Şahin et al. [78], Löytömäki et al. [42], and Sevincok et al. [43], which suggest that children with ADHD present obvious difficulties in understanding the false beliefs of others due to a deficit in mental attribution and reflection on complex social contexts. This is particularly evident in tasks that require the ability to consider multiple viewpoints simultaneously, such as second-order tasks [79].

This difficulty may be closely related to the increased impatience and impulsiveness that characterize many children with ADHD. These behavioral traits may hinder the ability to stop and reflect on social dynamics and to adequately assess the beliefs and intentions of others. The deficiency in the control of EFs, which is one of the defining features of ADHD, significantly impacts the ability to monitor attention during social situations, further complicating the understanding of false beliefs. The relationship between deficits in ToM and difficulties in EFs, such as attention control and inhibition of impulsive thoughts, is widely documented in the literature and suggests that improvement in these areas could lead to improvement in ToM skills as well [80].

In addition to the FBT, other significant instruments for evaluating ToM are the Faux Pas Recognition Test (FPT) and the Strange Stories Test. The FPT, which assesses the ability to recognize social mistakes such as misunderstandings or inappropriate behavior, has revealed that children with ADHD also have significant difficulties recognizing and understanding the emotions of others, such as shame or discomfort. These deficits are partly due to the difficulty in interpreting the social implications of a situation, which requires not only the ability to consider the emotions and intentions of others but a good deal of cognitive flexibility and metacognition. This aspect has been extensively explored in research such as that of Dagdelen [47], which found significantly lower scores in children with ADHD than in typical peers [81].

Besides difficulties in first- and second-order ToM tasks, poor ability to respond promptly to social cues in real time is another factor that jeopardizes the social integration of children with ADHD. The work of Hughes et al. [82] highlights how children with ADHD have greater difficulties in recognizing and reacting correctly to unexpected social situations, increasing the risk of dysfunctional social interactions. These difficulties, in fact, are not only cognitive but related to difficulty in managing social emotions, such as difficulty in inhibiting impulsive reactions, which could further compromise the interaction [46].

In regard to the Strange Stories Test, which measures understanding of others’ complex intentions, such as deception or mistaken beliefs, it has been confirmed that children with ADHD score lower than children with typical development. This difficulty in understanding the more sophisticated motivations of others is fundamental to social interactions and may prevent children with ADHD from grasping subtle social dynamics, such as the dissonance between what one person believes and what another person knows. The difficulty in attributing complex intentions and motivations to others is particularly pronounced in tasks requiring deeper thinking, which is closely linked to the self-control deficit and difficulties in executive performance that characterize ADHD [83].

The Interpersonal Reactivity Index (IRI), which measures affective and cognitive empathy, has also revealed that children with ADHD present a discrepancy between the two components. While affective empathy, which involves emotional influence from others, does not appear to be severely impaired, cognitive empathy, which requires the ability to understand the emotions and intentions of others, is lower than in TD children. This distinction may explain the phenomenon whereby children with ADHD may be emotionally involved in a situation but struggle to fully understand the dynamics of the situation, with the risk of responding inappropriately to crucial social cues [84].

In summary, although deficits in ToM in children with ADHD are now widely documented and recognized as one of the fundamental aspects of these children’s difficulty in social interactions, the implications of these findings for the implementation of rehabilitation interventions remain largely unclear and fragmented. The difficulty in understanding the beliefs, intentions, and emotions of others is a hallmark of the disorder, but the underlying mechanisms and the ways in which these deficits affect daily life and interpersonal dynamics are still not fully understood. Consequently, the practical application of scientific findings in the treatment of children with ADHD, although promising, is still far from providing unambiguous answers and systematic therapeutic solutions [35].

Difficulties in EFs, such as attention management, working memory, and inhibition of impulsive responses, appear to underlie ToM deficits in children with ADHD. Indeed, many of the social difficulties that children with ADHD encounter in everyday interactions, such as misunderstandings or inappropriate responses, can be attributed to difficulties in regulating attention or maintaining focus on what others are thinking or feeling. Working memory, which allows one to retain and manipulate information necessary to understand and anticipate the actions of others, is often impaired in children with ADHD. Furthermore, the tendency to act impulsively without reflecting on the effects of one’s actions makes the process of understanding the social intentions of others even more difficult. These difficulties in EFs could therefore represent a main target for therapeutic interventions, as intervening in these areas could not only improve the ability to manage one’s own actions but foster greater social awareness and a better understanding of interpersonal dynamics [85].

However, although the focus on cognitive functions is crucial, it is evident that a rehabilitation approach for children with ADHD cannot be limited to improving cognitive skills alone. An effective intervention should include a more integrated approach that also considers the emotional and metacognitive aspects of the disorder. Metacognitive strategies, which help children reflect on their own thought processes, could be particularly helpful for children to develop a greater awareness of their own cognitive, emotional, and social functioning. Similarly, it is crucial to introduce emotional strategies that can teach children with ADHD to recognize and manage their emotions in a more adaptive way, thus improving their ability to regulate impulsive responses and interact more effectively with others. Integrating these emotional and metacognitive components into treatments could significantly enhance children’s ability to understand not only cognitive dynamics but the emotions and social intentions of others [86].

In addition, a key aspect emerging from the research is the need to tailor therapeutic interventions, considering the individual variables that characterize each child with ADHD. Each child presents a unique profile of difficulties, which may vary depending on age, degree of symptom severity, comorbidities, and family and school context. Some children with ADHD may need interventions focused primarily on improving EFs, while others may benefit more from interventions that focus on improving social empathy or emotion regulation. Future research should address these gaps by developing treatment models that are responsive to the specific needs of each child. In particular, longitudinal studies should be conducted to explore how ToM deficits evolve over time and how different treatment modalities may affect their development in order to optimize interventions and improve their effectiveness [87].

The practical implications of these reflections are enormous: adopting a personalized approach that takes into account individual differences will not only make interventions more effective but will help reduce the social isolation that many children with ADHD experience. Indeed, improving social skills and emotional understanding can have a profound impact on the quality of children’s interpersonal interactions, reducing the risk of peer frustration and rejection, which often results in further isolation. A whole-person-based approach aimed at improving executive functioning while developing emotional and metacognitive skills could also lead to an improvement in the overall well-being of children with ADHD. This approach could facilitate their school adaptation, foster greater autonomy, and improve their self-esteem, reducing the psychological suffering resulting from the perception of feeling ‘different’ or ‘inadequate’ compared with peers [71].

## 5. Conclusions

Despite significant progress in understanding Theory of Mind (ToM) deficits in children with ADHD, research is still in an evolving phase, and many of the practical implications for designing effective therapeutic interventions still need further exploration. ToM deficits, which involve difficulties in interpreting and responding to others’ emotions and intentions, contribute to social isolation and challenges in interpersonal interactions. The practical applications of these findings suggest integrating specific interventions such as ToM training, role-playing, and educational programs to foster the development of social skills in children with ADHD. Additionally, it is crucial to involve families in psychopedagogical support programs to improve children’s emotional and social awareness. Future research should focus on more personalized approaches that consider not only cognitive difficulties but emotional and metacognitive components, such as emotional regulation and self-awareness. It will also be important to explore the effectiveness of innovative digital interventions and longitudinal studies to monitor the evolution of these deficits over time. In summary, to develop effective therapeutic interventions that improve the quality of life and social integration of children with ADHD, an integrated approach is needed that takes into account all cognitive, emotional, and social aspects, promoting long-term psychological well-being.

## Figures and Tables

**Table 1:** Overview of ADHD diagnostic criteria based on DSM-5-TR.

Category	Criterion	Description
Inattention	A. Attention to details	Often fails to give close attention to details or makes careless mistakes in schoolwork, work, or other activities.
	B. Sustained attention	Has difficulty sustaining attention in tasks or play activities (e.g., during lessons, conversations, or extended reading).
	C. Listening	Often does not seem to listen when spoken to directly (appears to be elsewhere mentally).
	D. Following instructions	Fails to follow through on instructions and does not complete schoolwork, chores, or workplace duties.
	E. Organization	Has difficulty organizing tasks and activities (e.g., managing time, keeping materials in order, meeting deadlines).
	F. Avoidance of sustained effort	Avoids or is reluctant to engage in tasks requiring sustained mental effort.
	G. Losing necessary items	Frequently loses objects necessary for tasks or activities (e.g., school supplies, keys, documents).
	H. Easily distracted	Is easily distracted by extraneous stimuli or unrelated thoughts.
	I. Forgetfulness	Often forgets daily activities (e.g., chores, appointments, bill payments).
Hyperactivity/Impulsivity	A. Motor restlessness	Often fidgets with hands or feet, squirms in seat.
	B. Leaving seat frequently	Gets up in situations where remaining seated is expected.
	C. Excessive movement	Runs or climbs in inappropriate situations (in adults, this may manifest as restlessness).
	D. Difficulty playing quietly	Struggles to engage in leisure activities quietly.
	E. Always “on the go”	Acts as if “driven by a motor,” has difficulty staying still.
	F. Excessive talking	Talks excessively, struggles to regulate speech.
	G. Impulsively answering	Answers before a question is completed, interrupts others.
	H. Difficulty waiting turn	Struggles to wait for their turn in social situations or games.
	I. Interrupting and intruding	Interrupts conversations or intrudes on others’ activities.
General diagnostic requirements	Persistence of symptoms	Several symptoms of inattention or hyperactivity-impulsivity persisted for at least 6 months at a level inconsistent with developmental stage and negatively impacting social, academic, or occupational activities.
	Early onset of symptoms	Several symptoms of inattention or hyperactivity-impulsivity were present before the age of 12.
	Symptoms in multiple settings	Symptoms are present in two or more settings (e.g., at home, school, or work; with friends or relatives; in other activities).
	Significant impairment	Clear evidence that symptoms interfere with social, academic, or occupational functioning or reduce quality of life.
	Exclusion of other disorders	Symptoms do not occur exclusively during schizophrenia or another psychotic disorder and are not better explained by another mental disorder (e.g., mood disorder, anxiety disorder, dissociative disorder, personality disorder, substance intoxication, or withdrawal).

**Table 2:** Main characteristics of the included studies.

Study	Population	Control Group	First Order Empathy Measure	Second Order Empathy Measure	Results
Löytömäki 2020 [42]	20 ASD (2 female) mean age 8.25 (SD = 1.21); 17 ADHD (3 female) mean age 8.06 (1.30); 13 DLD (4 female) mean age 7.62 (1.61)	TD: 106 (59 female). Mean age 8.02 SD = 1.42)	Sally–Anne task	Ice Cream Truck/Van Task	Children with ADHD exhibited notably lower ToM performance than TD group
Sevincok 2021 [43]	50 ADHD (7 female) age 10.00 (SD = 1.70)	40 TD (6 female) age 11.80 (SD = 2.00)	Sally–Anne task; RMET	NA	All ToM scores were significantly lower in children with ADHD than in TD children
Maoz 2017 [44]	24 ADHD (66% male) age 10.28 (SD = 1.64)	36 TD (53% male) 9.37 (SD = 1.35)	FPRT	IRI	Children with ADHD displayed reduced self-reported empathy
Özbaran 2018 [45]	100 ADHD (41 female) 14.03 (SD = 1.75)	100 TD (41 female) 14.03 (SD = 1.75)	RMET	UOT	ToM scores were lower in children with ADHD, and a significant link between ToM abilities (especially UOT) and emotion regulation was observed
Kılınçel 2021 [46]	42 ADHD (52.4% male) 13.20 (SD = 1.30)	41 TD (56.1% male) 12.10 (SD = 2.51)	FPRT, Smarties Test	II Order: Ice Cream Truck Test	ToM skills were impaired in adolescents with ADHD and were linked to the severity of the disorder
Dağdelen 2021 [47]	60 ADHD (30 female) 14 (SD = 1.43); 60 SD (30 female) 14.02 (SD = 14,44)	60 TD (30 female) age 13.55 (SD = 1.41)	FPRT, RMET, the Hinting Task	DERS	Adolescents with ASD and ADHD had weaker ToM and ER abilities than TD, and ToM impairments negatively affected ER in all groups
Singh 2021 [35]	20 ADHD 10.00 (SD = 2.29) (3 female); 20 SLD 11.45 (SD = 2.18)	20 TD 11.35 (SD = 2.51) 11.45 (SD = 2.18)	ToMI, ToM task battery	NA	Children with ADHD showed greater deficits in ToM tasks than children with SLD and TD of similar age and education
Cailles 2014 [48]	15 ADHD (5 female), mean age 9 (SD = 1.3)	15 TD mean age 9 (SD = 1.93)	NA	The ice cream story, the birthday story	Only 5 out of 15 children with ADHD succeeded in the second-order false-belief tasks, compared with 14 out of 15 controls
Razjouan 2023 [49]	52 ADHD (31 female) 14.05 (SD = 1.81)	42 TD (12 female) 14.32 (SD = 2.49)	Th.o.m.a.s.; RMET	NA	No significant differences in ToM abilities were found between the two groups, nor was any correlation found between ToM scores and sociocultural factors
Parke 2018 [50]	25 ADHD (70% male) 10.57 (SD = 2.09)	25 TD (60% male) 10.07 (SD = 1.90)	Happé’s Strange Stories; IRI; NEPSY II; RMET	NA	Children with ADHD performed worse on cognitive ToM, affect recognition, and cognitive empathy than TD peers
Bozkurt 2024 [51]	47 ADHD 10.0 years (SD = 1.7) (9 female)	38 TD 10.6 (SD = 1.8) (10 female)	RMET; FPRT	NA	The recognition of disgust and ToM skills were positively correlated, suggesting distinct deficits in social cognition related to emotion recognition
Lee 2014 [52]	14 ADHD (14 male) 13.37 years (SD = 0.90)	19 TD (11 male) 13.35 (SD = 1.18)	IRI; EQ-C-CEE; EQ-C-CE	NA	The ADHD group had a smaller cortical volume linked to emotional empathy than the control group, with no brain region showing a significant correlation with empathy
Rumpf 2012 [53]	11 ADHD (1 female); 9.11 years, (SD = 11.8) 11 AS (all males) 10.5 years, (SD = 16.9)	11 TD (1 female) 9.11 years, (SD = 11.8)	Telling a story from a book	NA	Children with ADHD had ISL use similar to HC, suggesting no significant impairment in emotional empathy
Mary 2015 [33]	30 ADHD 10.3 years (SD = 1.28)	31 TD 10.0 years (SD = 1.0)	RMET; FPRT	NA	ToM deficits in children with ADHD were primarily due to impairments in attention and EFs, which may contribute to their socioemotional difficulties
Mohammadzadeh 2016 [54]	30 ADHD 7.70 years, (SD = 1.77)	30 TD 9 years (SD = 1.94)	Moving shapes paradigm task (or animated triangles)	NA	Children with ADHD performed significantly worse than normal children (*p* < 0.05) in comprehending others’ intentionality
Yilmaz Kafali 2021 [55]	15 ADHD, 13.9 ± 1.8 years (77 male).		RMET	NA	Adolescents with ADHD who were victims or perpetrators of bullying demonstrated poorer ToM abilities than those who were not involved in bullying
Demurie 2011 [56]	13 ADHD (1 female) 13.69 (SD = 1.43) 13 ASD (1 female) 14.35 (SD = 1.24)	18 TD (4 female) 13.86 (SD = 1.73)	IRI, RMET	EAT	Adolescents with ADHD showed similar empathic accuracy to those with ASD and controls, suggesting mind-reading deficits potentially linked to inhibitory control issues
Mohammadzadeh 2019 [57]	ADHD 30 7.28 (SD = 1.64)	30 TD 7 years, (SD = 1.34)	Animated Triangles Task	NA	ADHD group had a significant ToM and EF impairment relative to the TD group
Bolat 2017 [58]	ADHD 69 (21 females), 10.17 years (SD = 1.99).	69 TD (21 females), 10.28 years (SD = 2.10).	First- and second-order false belief Tasks; CT	UOT	ADHD had an independent negative effect on overall ToM and emotion recognition abilities
Yang 2009 [59]	ADHD 26 participants (4 females), 8.2 years (SD = 2.9). ASD 20 (2 females) 8.1 years (SD = 3.5)	30 TD participants (3 females) 8.0 years (SD = 3.1).	Appearance–Reality Task, Unexpected-Location Task	Unexpected-Content Task	Children with ADHD performed similarly to TD children on ToM tasks, showing no significant deficits in first-order or second-order ToM
Perner 2002 [60]	ADHD 24 children, 67.0 months (SD = 7.1).	22 TD 69.4 months (SD = 7.1).	NA	Second-order false belief task	The at-risk ADHD group performed worse than the control group on several executive tasks but showed no impairment on the advanced Tom tasks
Pitzianti 2017 [3]	ADHD group: 23 (9 females), 10.39 years (SD = 2.35)	20 TD (9 females) 9.10 years (SD = 1.92).	NEPSY-II	NA	The study found no significant differences in ToM or emotion recognition performance between children with ADHD and TD, suggesting that ToM and ER deficits were not a consistent feature in ADHD
Berenguer 2018 [61]	ADHD 26, 9.96 years (SD = 14.93) ASD 19, 9.68 years (SD = 24.63)	30 TD 9.23 years (SD = 12.06)	ToMI; NEPSY-II	NA	Children with ADHD showed impairments in ToM tasks, performing better than those with ASD but worse than those TD, especially in applying ToM knowledge to real-life situations

Legend: ASD: autism spectrum disorder; ADHD attention deficit hyperactivity disorder; DLD: developmental language disorder; TD: typically developing; ToM: Theory of Mind; RMET: Reading Mind in the Eyes Test; FPRT: Faux Pas Recognition Test; IRI: Interpersonal Reactivity Index; UOT: Unexpected Outcomes Test; DERS: Difficulties in Emotion Regulation Scale; ER: emotion regulation, EQ-C-EE: emotional empathy, EQ-C-CE: cognitive empathy; AS: Asperger syndrome; ISL: internal state language, EAT: empathic accuracy task, CT: Comprehension Test; ER: emotion recognition; EFs: executive functions; NA: not available.

**Table 3:** Strengths and limitations of the tests used.

Test	Strengths	Limitations
False Belief Task (FBT)	Widely used and well-established; detects impairments at both first- and second-order levels; sensitive to difficulties in understanding others’ beliefs and intentions [62].	Some studies have shown inconsistent results in second-order tasks; the relationship with executive functions (EF) may vary [62].
False Passage Recognition Test (FPRT)	Effectively evaluates social cognition and ToM deficits [63]; can highlight correlations with symptom severity (e.g., inattention, impulsivity) [63].	Variability related to age and sample size [63].
Happé’s Strange Stories	Highlights difficulties in interpreting complex social interactions and subtle intentions [64].	Focus on complex scenarios that might limit generalizability [50].
Narrative and Internal State Language (ISL)	Helps distinguish deficits in narrative coherence [65].	Grammatical skills may remain intact, masking difficulties [53].
NEPSY-II	Allows investigation of the relationship between executive functions and ToM [66].	Variable results in subtests due to discrepancies in emotion recognition and the relationship with executive functions [66].
Reading the Mind in the Eyes Test (RMET)	Complementary data (e.g., eye-tracking) highlight differences between ADHD and TD groups [67].	Possible confusion between visual attention and ADHD symptoms; variations based on the age of the sample [67].
Theory of Mind Assessment Scale (Thomas)	Effective for assessing ToM and EF and for group comparisons [68].	Has not always shown significant differences between children with ADHD and TD [68].
Animated Triangles Task	Detects deficits in attributing mental states to abstract stimuli [69].	Correlation with executive functions is not always clear [69].
Empathy Quotient (EQ)	Investigates the cognitive dimensions of empathy and highlights deficits in perspective-taking [41].	Risk of self-report bias [41].
Theory of Mind Inventory, TOM Battery	Comprehensive approach covering early, basic, and advanced levels of ToM; useful for differentiating ADHD, DSA, and TD [35].	Complex administration; possible caregiver report bias [35].
ToMI (Theory of Mind Inventory)	Revised version that assesses ToM through parent reports; highlights differences between groups (ADHD, ASD, TD) and covers various ToM abilities [70].	Difficulty in clearly separating ToM deficits from those of EF [70].

## Data Availability

Not applicable.
